# The Omicron XBB.1 Variant and Its Descendants: Genomic Mutations, Rapid Dissemination and Notable Characteristics

**DOI:** 10.3390/biology13020090

**Published:** 2024-02-01

**Authors:** Raffaele Giancotti, Ugo Lomoio, Barbara Puccio, Giuseppe Tradigo, Patrizia Vizza, Carlo Torti, Pierangelo Veltri, Pietro Hiram Guzzi

**Affiliations:** 1 Department of Surgical and Medical Sciences, Magna Graecia University of Catanzaro, 88100 Catanzaro, Italy; raffaele.giancotti@unicz.it (R.G.); ugo.lomoio@unicz.it (U.L.); barbara.puccio@unicz.it (B.P.); vizzap@unicz.it (P.V.); torti@unicz.it (C.T.); 2 SMARTEST Lab., eCampus University, 22060 Novedrate, Italy; giuseppe.tradigo@uniecampus.it; 3 Department of Computer Engineering, Modelling, Electronics and System, University of Calabria, 87036 Rende, Italy; pierangelo.veltri@dimes.unical.it

**Keywords:** XBB variant, Omicron, COVID-19, genomic analysis

## Abstract

**Simple Summary:**

SARS-CoV-2 XBB variant was isolated in Singapore in 2022. Sequence changes and their relation with spike protein structure is studied with respect to XBB subvariants. Structural and functional distinctions of the variants is also reported. Affonity binding between the spike protein and ACE2 is reported. The relation among sequence and structure has been studied.

**Abstract:**

The SARS-CoV-2 virus, which is a major threat to human health, has undergone many mutations during the replication process due to errors in the replication steps and modifications in the structure of viral proteins. The XBB variant was identified for the first time in Singapore in the fall of 2022. It was then detected in other countries, including the United States, Canada, and the United Kingdom. We study the impact of sequence changes on spike protein structure on the subvariants of XBB, with particular attention to the velocity of variant diffusion and virus activity with respect to its diffusion. We examine the structural and functional distinctions of the variants in three different conformations: (i) spike glycoprotein in complex with ACE2 (1-up state), (ii) spike glycoprotein (closed-1 state), and (iii) S protein (open-1 state). We also estimate the affinity binding between the spike protein and ACE2. The market binding affinity observed in specific variants raises questions about the efficacy of current vaccines in preparing the immune system for virus variant recognition. This work may be useful in devising strategies to manage the ongoing COVID-19 pandemic. To stay ahead of the virus evolution, further research and surveillance should be carried out to adjust public health measures accordingly.

## 1. Introduction

The SARS-CoV-2 genome is composed of 29.9 kilobases [1] and has 14 open reading frames (ORFs). It contains multiple sections that encode four structural proteins: spike (S), envelope (E), membrane (M), and nucleocapsid (N). Furthermore, it is characterized by 16 non-structural proteins (nsp1-nsp16 complexes) and accessory proteins [2,3].

**Figure 1:**
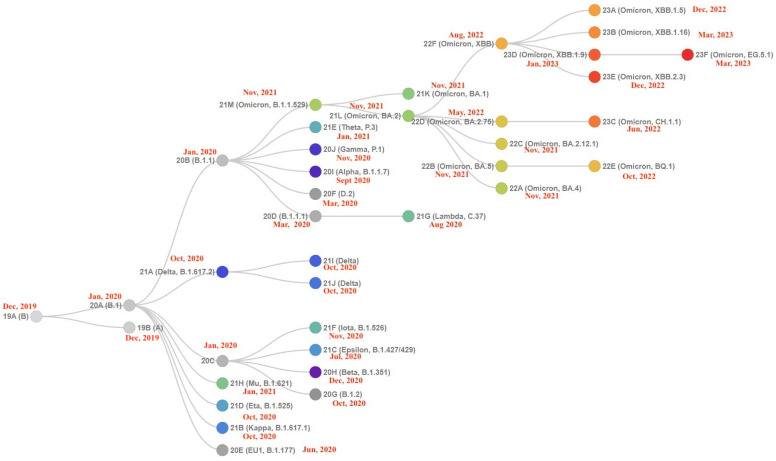
Phylogenetic relationships of SARS-CoV-2 clades. Covariants follow the Nextstrain Clade schema, where variants can descend from other variants. Starting from this figure, it is possible to show how the Omicron variant (21M clade, B.1.1.529) gave rise to a greater number of VOI/VOC. The tree has been generated from https://covariants.org (accessed on 29 January 2024). The red labels represent the date of first detection of each variant. As highlighted, XBB.1 showed a greater number of subvariants in a relatively short time.

SARS-CoV-2, like other viruses, undergoes several mutations during the replication process [4,5,6] due to errors in replication steps and modifications to the structure of the viral proteins [7]. Mutations determining competitive advantages of the associated virus are preserved [8,9]. For this reason, the World Health Organization (WHO) closely monitors SARS-CoV-2 mutations, as reported in Figure 1. Among its activities, the WHO is responsible for selecting variants that may require the attention of government public health services to rapidly define guidelines and actions to contain viral evolution [10,11,12]. Variants with similar genetic changes and/or shared attributes are indicated as a Variant Being Monitored (VBM), a Variant of Concern (VOC), or a Variant of Interest (VOI). The Omicron variant represents an important milestone in the evolution of SARS-CoV-2 variants, mainly due to its high mutation rate. It has been proven that Omicron’s high number of mutations makes it more contagious than earlier versions [13]. Moreover, it seems to be better equipped to avoid the immune system’s response to prior infection or immunization. Nevertheless, the Omicron variant is usually milder than its predecessors; hence, the risk of severe illness or death is much lower [5,14]. The development of the Omicron variant has been intricate and ever-changing. We focus here on the more recent evolution of the virus, also indicated as the XBB family [15]. The original Omicron variant (BA.1) was the leading variant for a few months, later to be replaced by many subvariants, such as BA.2, BA.4, and BA.5. These subvariants were thought to be more contagious than BA.1, but they did not appear to pose such a threat to the human defence mechanism.

The current wave of COVID-19 cases (at the time of the writing of the manuscript, September 2023) is being driven in many countries by the BA.5 subvariant. BA.5 is believed to be even more transmissible than BA.4 and is more likely to evade the immune response from prior infection or immunization. Fortunately, the risk of severe illness and death from BA.5 is still relatively low [16,17].

**Figure 2:**
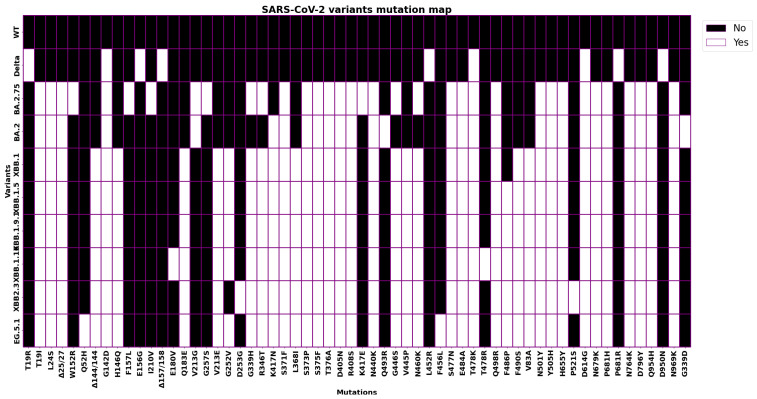
The matrix reports the mutations of Delta B.1.617.2, Omicron BA.2, and XBB variants on the S protein. All the XBB descendants share almost all the XBB mutations. EG.5.1 presents two unique mutations, Q52H and F562L.

The Omicron XBB variant [18,19] is a subvariant of the Omicron variant BA.2.75, which was first identified in South Africa (December 2022) and has been detected in other countries, including the United States, Canada, and the United Kingdom. The XBB variant is believed to be more transmissible than the original Omicron variant, as well as more likely to evade the immune response from previous infection or vaccination. It has 32 mutations, including 10 mutations in the spike protein. Figure 2 reports a summary of the mutations limited to spike. It may be more likely to crossover the immune response for previous infections or vaccinations [20].

**Figure 3:**
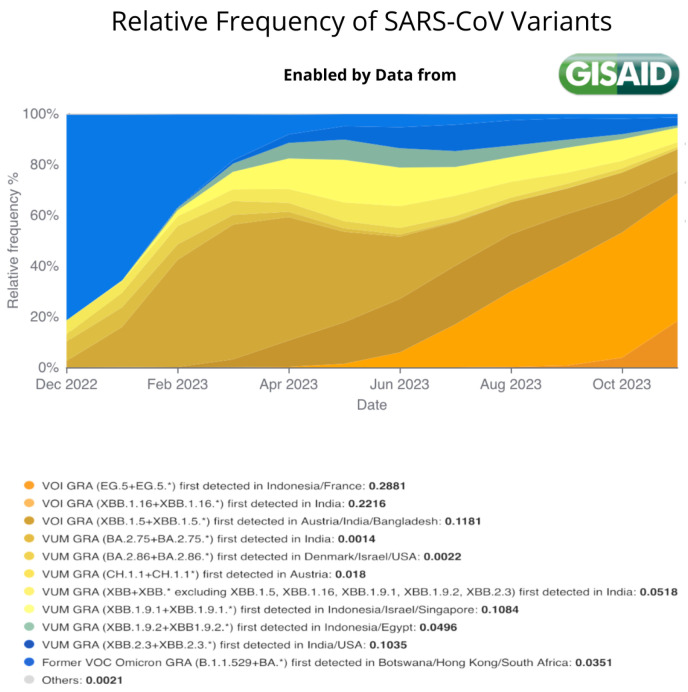
Figure shows the relative frequency of the SARS-CoV-2 variants from January 2023 until September 2023. It highlights the relatively rapid evolution of the virus and the rise of EG.5.1 variant since May 2023. Data extracted from https://gisaid.org/hcov-19-variants-dashboard/ [22] (accessed on 28 November 2023).

At the time of writing (September 2023), XBB has continued to evolve, yielding to the appearance of XBB1.5, XBB1.16, XBB1.91, and EG5.1 subvariants. Figure 3 describes the number of infected people and the related variants [21]. Their distribution at a national level is reported in Appendix A. We consider such data as the global scenario of the XBB subvariants at present.

We consider questions such as the following: (i) Is the pattern of the evolution of XBB different from the overall pattern of the evolution of SARS-CoV-2? (ii) Do XBB and its descendents present any peculiar characteristics that may determine a new outbreak?

We study the XBB lineage, characterizing XBB spike protein mutations with respect to the ones present in previous variants. We study EG.5.1 mutations and show how the EG.5.1 variant presents differences in terms of net charge and binding affinity with respect to the descendants of XBB.

### The Landscape of the Omicron Variants

We consider the Omicron variants as the evolution of the XBB variant studied here, which is a subvariant of BA.2. BA.2 can be considered as a BA.1 subvariant containing some unique receptor binding domain (RBD) spike mutations. The T376A, D405N, and R408S substitutions in the strategic antigenic site are associated with its capacity to evade immunization and high transmissibility.

XBB, identified for the first time in 2022 in Singapore, is a recombinant of two Omicron sublineages: (i) BJ.1 (also indicated as BA.2.10), called *Argus*, and (ii) BM.1.1.1 (also indicated as BA.2.75) called *Mimas*. It quickly began to spread throughout the world. XBB was considered to be the most immune-evasive COVID variant at the time, surpassing the immune-evasiveness of BA.5, which was dominant worldwide until the end of August 2021. The XBB variant presents a strong capacity for crossing over the immune system.

The XBB variant, also named *Gryphon*, started to dominate the SARS-CoV-2 scene, and the majority of the circulating variants are now XBB descendants (also known as the *Gryphon Family*). As reported in Figure 1, XBB descendants (see node 22f; Omicron, XBB in the descendant tree) can be summarized as follows:

- XBB.1.5 (Kraken) emerged due to a genetic recombination between two BA.2 sublineages (see ancestors of XBB nodes in the tree) combined with S486P mutations at a significant point in its evolutionary history.
- XBB.1.9.1 (Hyperion) is XBB.1.5’s sibling.
- XBB.1.16 (Arcturus) was initially identified in India with a single mutation (K478R) in the RBD of XBB.1.5. Earlier studies demonstrated that mutations K417N, Q498R, and N501Y in the RBD region increase the ability of the variant to bind to the human ACE2 receptor. Mutations in residue 484 in the loop area have been associated with the virus’s ability to evade the immune system.
- XBB.2.3 (Acrux) first appeared in late December 2022 in India, even though it did not begin to spread until March 2023. It presents a highly evasive mutation, S:T478K.
- EG.5.1 (Eris) is a direct descendent of XBB.1.9.2, which has the same spike amino acid profile as XBB.1.5. EG.5.1 was first reported in February 2023 and designated as a variant under monitoring (VUM) on 19th July 2023 [23].

Figure 2 shows the mutationsDelta B.1.617.2, Omicron BA.2, and the subvariants of the XBB Omicron, considering only mutations of the S protein. Figure 3 shows the relative frequencies of the detected cases from January to September 2023. The detailed mutation landscape across the whole viral genome is reported in Appendix A.

We focus on the XBB EG.5.1 descendant and compare it with previously identified variants and active ones. We also pay attention to the evolutionary mutations, speed of variant diffusion, and virus activity with regards to the spread of infection.

## 2. Materials and Methods

To study the XBB family, we analyzed the variants to verify binding affinity among ACE2 and the studied variants [1]. We studied the variants to determine the impact of variants on the structure of the spike proteins of each variant and to characterize some phenotypical properties. The relation between sequence and structure was analyzed using a parametric Pearson test. We analyzed the correlation between sequence distance and structure distance. Structural distances were measured by calculating the TM-scores [24] between pairs of spike proteins of two different variants by using the Universal Structural alignment (US-align) software [25]. Sequence distances were measured using the CLUSTALW software settings parameters at the default values [26]. The significance was assessed using the false discovery rate (FDR) measure for multiple testing. An FDR lower than 0.01 was considered significant.

We examined the Omicron structure subvariants of the SARS-CoV-2 XBB spike glycoprotein in 3 conformations: (i) spike glycoprotein in complex with ACE2 (1-up state) (PDB code 8IOU), (ii) spike glycoprotein (closed-1 state) (PDB code 8IOS), and (iii) spike glycoprotein (open-1 state) obtained by removing ACE2 from PDB code 8IOU. Each variant with its mutations is shown in Table 1. Sequence data were downloaded from the PDB [27], since it also provides such data.

Structural data of the spike protein of the XBB.1 variants were also downloaded from the PDB database. We used the 8IOS structure to model the S protein in closed form and the 8IOU for the human ACE2–SARS-CoV-2 S complex. The open configuration of the S protein was obtained by removing human ACE2 from the complex. The 8IOS structure has a resolution equal to 2.50 Angstrom, while the structure of 8IOU has a resolution equal to 3.18 Angstrom. Both structures were determined using electron microscopy.

We used the *mutagenesis* tool of the PyMoL suite [28] for calculating all protein structures used in this work, starting from the PDB structures of XBB.1. We selected all the default parameters for mutagenesis. Such a tool selects the right rotamers by sorting the rotamers according to their frequencies of occurrence in proteins.

The binding affinity of the spike proteins of the variants and human ACE2 was calculated using PRODIGY, a web server that calculates the binding affinity of protein–protein complexes [29], available at https://wenmr.science.uu.nl/prodigy/, (accessed on 29 January 2024). We set the environment temperature at 36 degrees Celsius. For each complex, PRODIGY calculated the Δ*G*, i.e., the Gibbs free energy, and the $K_{d}$ dissociation constant.

For each variant, we computed the acid dissociation constant $pK_{a}$ for each amino acid of the analyzed proteins using the PROPKA3 web server [30]. Given a node, the value of $pK_{a}$ is equal to $-log_{10}K_{a}$, where $K_{a}$ is the acid dissociation constant that measures the acidity or alkalinity of the amino acid. Following this method, XBB subvariants’ evolution and pKa values were used to predict the overall domain charge.

## 3. Results

We investigated the relationship between sequence and structure distances. We analyzed the relationship between sequence and structure to characterize the XBB subvariants’ evolution.

**Figure 4:**
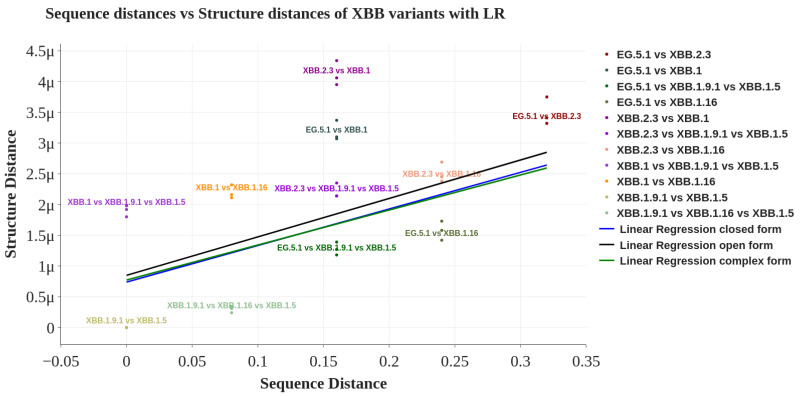
Each point in the figure represents the correlation of a pair sequence/structure. The XBB variants are considered in the closed conformation. The *x*-axis represents the distance between the sequences, while the *y*-axis represents the distance between the structures. The lines represent the linear regression between sequence distance and structure distance for open form, closed form, and complex.

The *x*-axis of the graph displays the pair-wise sequence distances calculated on the primary structure, while the y-axis reflects the pair-wise mutual distances of the protein structures. Each point on the graph represents the correlation between a pair of sequences and the structure distance [31]. The figure shows no correlation between sequence and structure distances, confirming the similarity in evolution between XBB descendants and the whole SARS-CoV-2 phylogeny. Figure 4 reports the relationships among structure and sequence distances of the XBB variants. By calculating the linear regression (the blue straight line reported in the figure), we obtained a low correlation index (i.e., an R-squared score equal to 0.213), which showed no evidence of a structural relationship between sequence and structure. The measure of the significance of the correlation is reported in Table 2, where both corrected false discovery rates [32] indicate a nonsignificant evidence of correlation between sequence and structure below the statistical significance threshold of 0.01. We conclude that these correlation analyses report a moderate but not strongly statistically significant correlation between XBB protein structure (in closed, open, and wild typeorm) and sequence. Interestingly, there is no difference in terms of general evolution of the spike protein, as analyzed in [13], where no correlation among sequence and structure was reported.

This result implies that structural (and thus functional) distinctions are highly dependent on the local structural context when examined at a finer level of detail, making it impossible to extrapolate the same information from the sequences.

We estimate the transmissibility of the affinity binding between the spike protein of the variants and the human ACE2 receptor by measuring the biochemical properties of the proteins.

**Figure 5:**
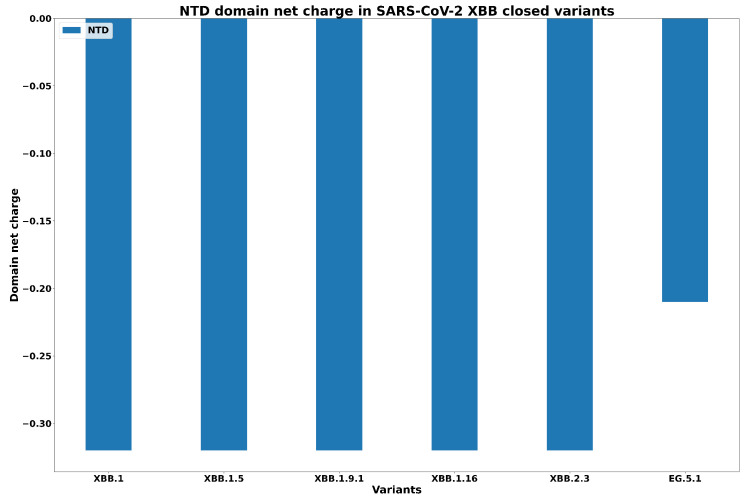
Net charges in the NTD domain for all XBB variants in closed conformation.

First, in Figure 5 we report the net charge of N-terminal domain (NTD) in each XBB.1 variant. We report a negative charge in all variants, indicating affinity to bind to human ACE2. We report similar values for all the XBB.1 subvariants except for EG.5.1 due the specific mutations of these variants. Surprisingly, EG.5.1 is more similar to Omicron than to XBB when considering the net charge of the NTD domain.

**Figure 6:**
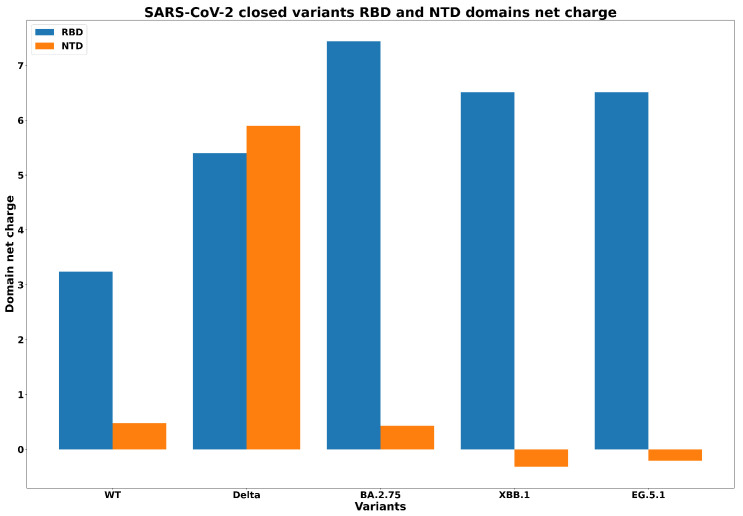
Net charges in the NTD and RBD domains for wild type, Delta, (Omicron) BA.2.75, XBB.1, and EG.5.1 variants in closed conformation.

Figure 6 shows, in a histogram format, the net charge in the NTD and RBD domains for each spike variant, including wild type form (with no mutations), Delta B.1.617.2, Omicron BA.2.75, XBB.1, and EG.5.1. The figure shows a considerable increase in net charge of the NTD domain from wild type to Delta variants and a considerable decrease from Delta to BA.2.75, which results in turn in the change in sign (from positive to negative) of the charge of the domain. In fact, mutations passing from BA.2.75 to XBB.1 and EG.5.1 have inversion versus negative values for NTD net charges. We can also see that the EG.5.1 variant is similar to XBB.1 in terms of RBD net charge, but they are different from the other precedent variants.

**Figure 7:**
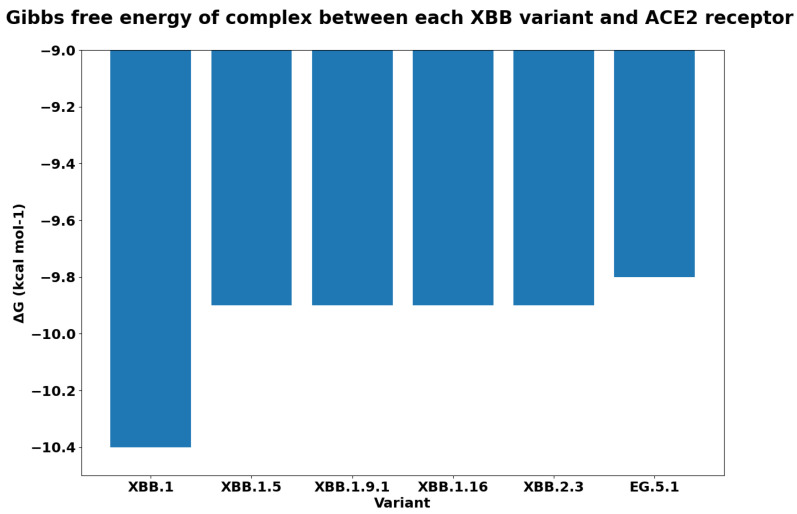
Gibbs free energy Δ*G* to quantify the binding affinity of each XBB variant with the ACE2 receptor.

**Figure 8:**
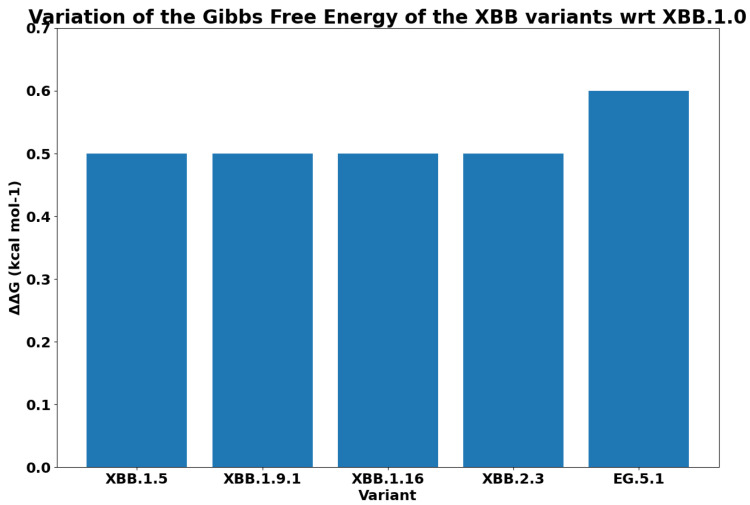
Figure reports differences between the Gibbs free energy of XBB with respect to its descendants (indicated as ΔΔ*G*).

**Figure 9:**
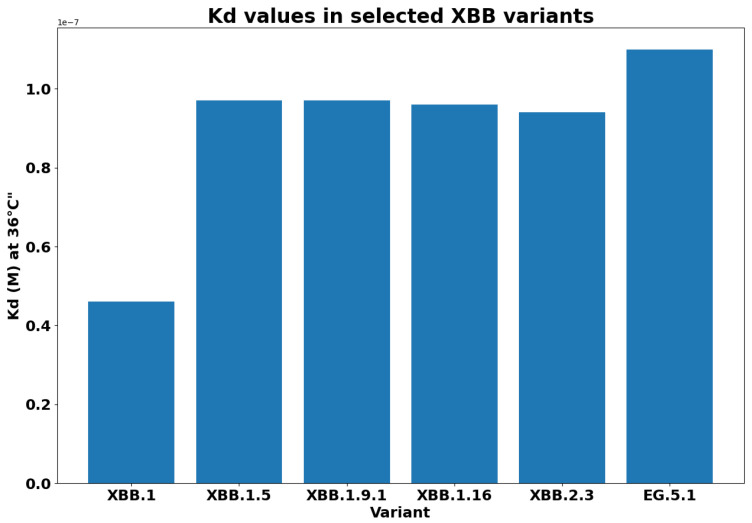
Dissociation constant $K_{D}$ for XBB descendant variants. A higher value of $K_{D}$ determines a stronger binding affinity. Note that EG.5.1 has the greatest binding affinity to the ACE2 receptor.

**Figure 10:**
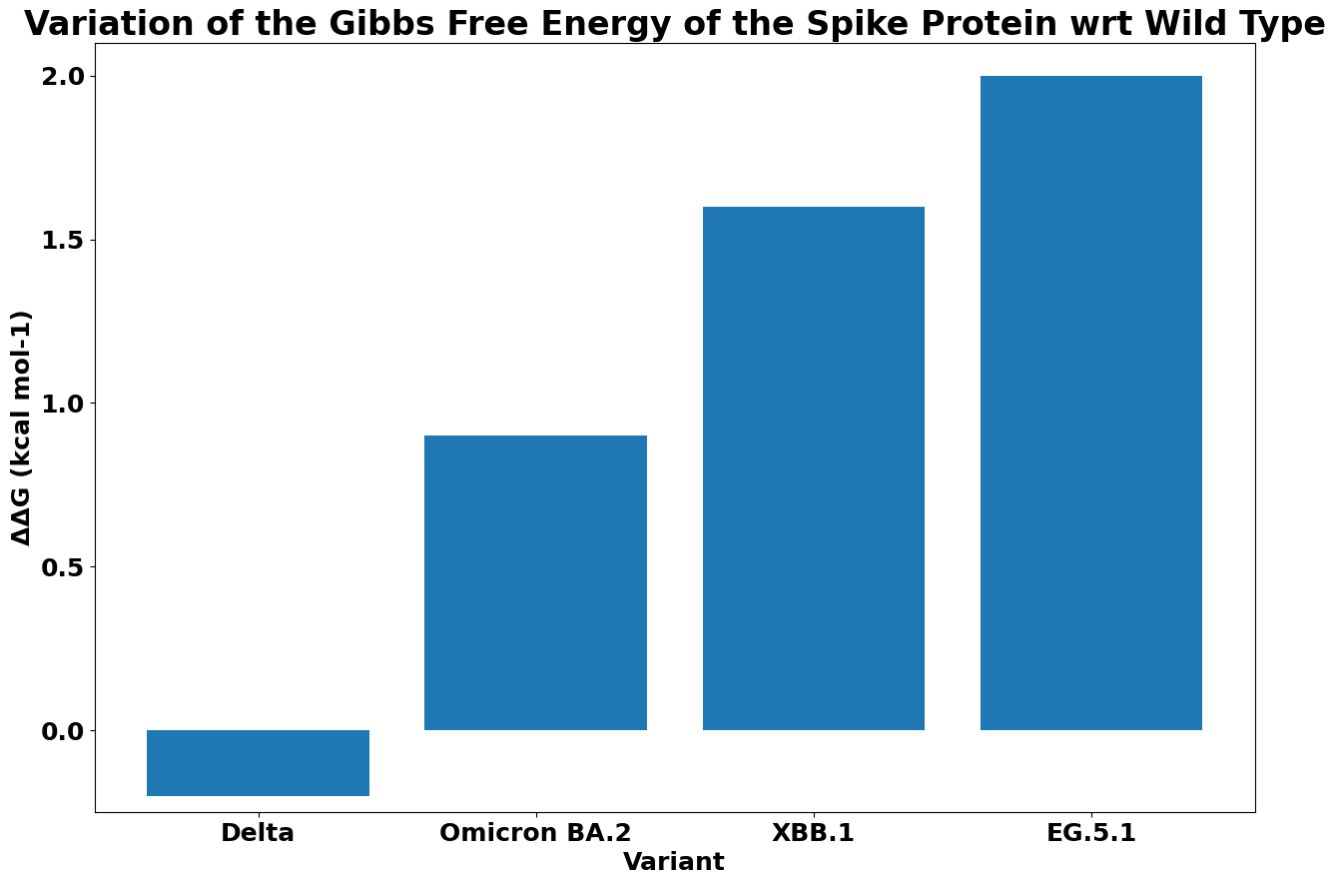
Gibbs free energy difference ΔΔ*G* between Delta, BA.2 Omicron, XBB.1, and EG.5.1 variants with respect to wild type.

For each variant, Figure 7 reports the Gibbs free energy. The subvariants of XBB have a lower ΔG, and this implies a greater binding affinity. As was expected, EG.5.1 has the maximum binding affinity. We reported the difference between the free energy of each variant and the XBB in Figure 8. We measured the ΔΔG as the difference among the calculated values of ΔG. We compared the binding affinity of some selected variants with respect to Omicron, and the results are reported in Figure 9. Similarly, we evaluated the binding affinity among Delta with respect to other variants, and the results are reported in Figure 10. Both figures report on energy and variation (i.e., ΔΔG). The characteristics of EG.5.1 are statistically significant when compared to other variants included in the analysis. The *p*-value obtained from the Wilcoxon test [33] is 0.0001.

## 4. Discussion

Starting from the branches reported in Figure 1, we focus on mutations that are common to variants. Note that the two mutations T478K and D614G are shared between XBB sub-variants (XBB.1 and EG.5.1) and variants from other branches (Delta and Omicron BA.2). This allowed us to verify if the mutations in XBB and ECG occur in other branches. The table reported in Figure 2 (also reported in the Appendix A in Table A1) shows the mutations overlapping among variants. Note that it is also possible to evaluate the affinity of the binding of variants with respect to ACE2 and the importance of mutations in different branches. Thus, we evaluated the free energy differences among the wild type and each considered mutation shared among variants (the evaluated values are reported in the Appendix A in Table A2). The energy difference between each variant with respect to the common mutation (i.e., both, T478K and D614G) has low values; thus, the variants present the same binding affinity with ACE2. Gibbs free energy and differences can be identified by considering Figure 10 (i.e., the ΔΔ*G* between the Delta variant and wild type reference is less than zero negative).

Compared to previous ones, the evolution of the XBB variant is interesting in terms of its speed and the number of cases identified in a short time [34]. XBB and XBB.1 have shown the highest levels of immune escape of all the Omicron sublineages currently identified and have shown significant reductions in the capacity of infecting vaccinated individuals. In particular, several substitutions of XBB.1 (the first descendent of XBB) have been shown to confirm significant resistance to BA.2 infections.

In the XBB mutations, spike protein variations are similar in terms of speed with respect to the ones present in previous variants. The EG.5.1 variant is radically different from the others and seems to spread much faster than previous variants, probably due to the fact that the number of real cases is greater than the recorded ones. Mutations of EG.5.1 make this variant more similar to the original Omicron (in particular Q52H and F456L). This implies a lower net charge and a greater binding affinity with respect to XBB descendants.

The XBB.1 variant presents many substitutions in the S protein that may cooperatively contribute to its resistance to immunity, which seems to be more resistant than the BA.2 variant [35]. This may explain the relatively high frequency of people reinfected by the XBB variant (and its descendants) [36]. Moreover, XBB.1.5 has shown an RBD spike mutation (F486P) that increases infectivity due to increased binding affinity to the angiotensin-converting enzyme 2 (ACE2) receptor [37]. Similarly, the EG.5.1 variant has shown increased prevalence, growth advantage, and immune escape properties. This is mainly due to the flip mutations F456L and L455F. Such interesting substitutions (nicknamed FLip-FLop) are of interest for two adjacent amino acids (455–456) of the RBD spike protein.

Focusing on XBB variants and their genomic sequences and protein structures, the results suggest that the evolution of such variants is similar to the overall SARS-CoV-2 phylogeny. This implies that the structural and functional distinctions depend on the context. The strong binding affinity for some of the XBB variants (as for EG5.1) raises questions on how to control the spread of these variants. These results may be relevant for studies related to the transmissibility and infectivity of these variants [38,39].

## 5. Conclusions

The manuscript focuses on XBB lineage variants, on changes in protein sequences and structures, and ACE-2 binding affinities in SARS-CoV-2. The paper is based on available datasets and highlights the necessity of acquiring data from COVID-19-positive cases to study and monitor the virus mutations. Future work may consider studies on surveillance effects useful to calibrate public health measures.

## Figures and Tables

**Table 1:** Mutations in the SARS-CoV-2 XBB spike variants. Mutations of Omicron BA.2 (clade 21L), Delta (clade 21A), and of the descendent of XBB.1.0 (considered as XBB.1.0) are reported.

Spike Variant	Mutations
Wild Type	No Mutations
Delta B.1.617.2	T19R + G142D + E156G + DEL157/158 + L452R + T478K + D614G + P681R + D950N
Omicron BA.2	T19I + L24S + DEL25/27 + G142D + V213G + G339D + S371F + S373P + S375F + T376A + D405N + R408S + K417N + N440K + S477N + T478K + E484A + Q493R + Q498R + N501Y + Y505H + D614G + H655Y + N679K + P681H + N764K + D796Y + Q954H + N969K
Omicron BA.2.75	T19I + L24S + DEL25/27 + G142D + W152R + F157L + I210V + V213G + G257S + G339H + R346T + S371F + S373P + S375F + T376A + D405N + R408S + K417E + N440K + G446S + N460K + S477N + T478K + E484A + Q498R + N501Y + Y505H + D614G + H655Y + N679K + P681H + N764K + D796Y + Q954H + N969K
XBB.1.0	T19I + L24S + DEL25/27 + V83A + G142D + DEL144/144 + H146Q + Q183E + V213E + G252V + G339H + R346T + L368I + S371F + S373P + S375F + T376A + D405N + R408S + K417N + N440K + V445P + G446S + N460K + S477N + T478K + E484A + F490S + Q498R + N501Y + Y505H + D614G + H655Y + N679K + P681H + N764K + D796Y + Q954H + N969K
XBB.1.9.1	XBB.1.0 + F486P
XBB.2.3	XBB.1.0 + V252G + D253G + F486P + P521S
XBB.1.5	XBB.1.0 + F486P
XBB.1.16	XBB.1.0 + E180V + T478R + F486P
EG.5.1	XBB.1.0 + Q52H + F456L + F486P

**Table 2:** Pearson correlation coefficients, along with related *p*-values and false discovery rate (FDR)-corrected *p*-values elucidating the connections between sequence and structure distances for the closed, open, and complex forms with ACE2 of the XBB variants spike protein. The significance was assessed by using the FDR *p*-value correction for multiple testing. A corrected *p*-value lower than 0.01 was considered significant.

Spike Structure	Pearson Coefficient	FDR Corrected *p*-Value
Closed	0.462	0.042
Open	0.447	0.042
Complex	0.447	0.042

**Figure A1:**
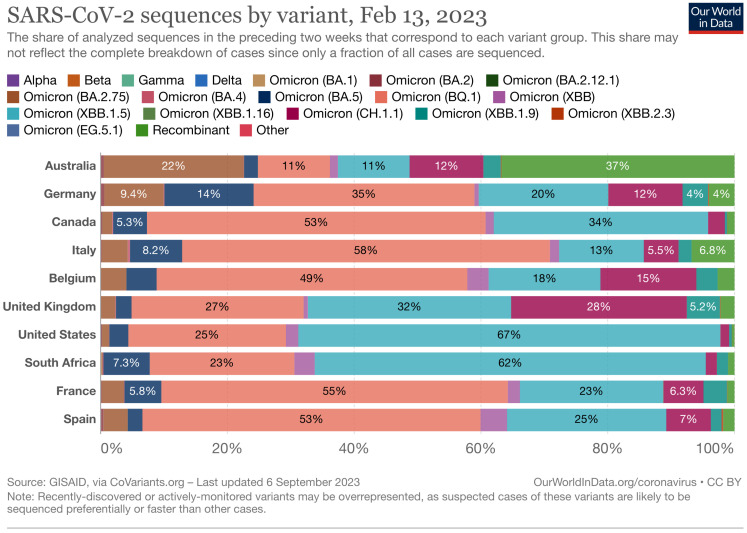
Relative frequency of Omicron variants in February. Image generated from https://ourworldindata.org/ (accessed on 29 January 2024) under open access under the Creative Commons BY license.

**Figure A2:**
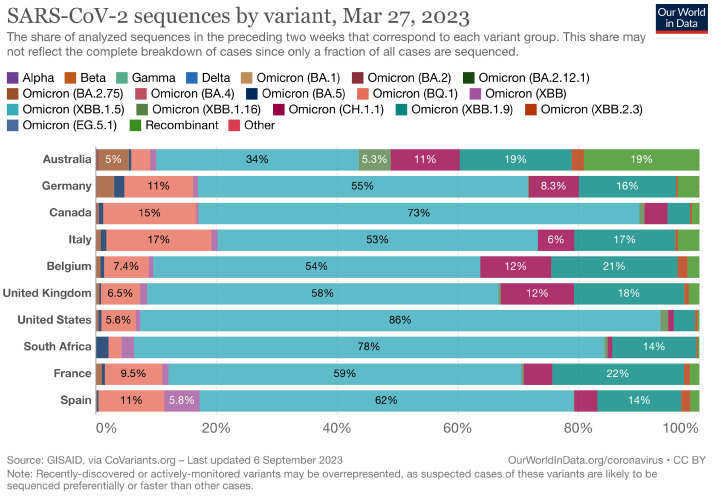
Relative frequency of Omicron variants in April 2023. Image generated from https://ourworldindata.org/ (accessed on 29 January 2024) under open access under the Creative Commons BY license.

**Figure A3:**
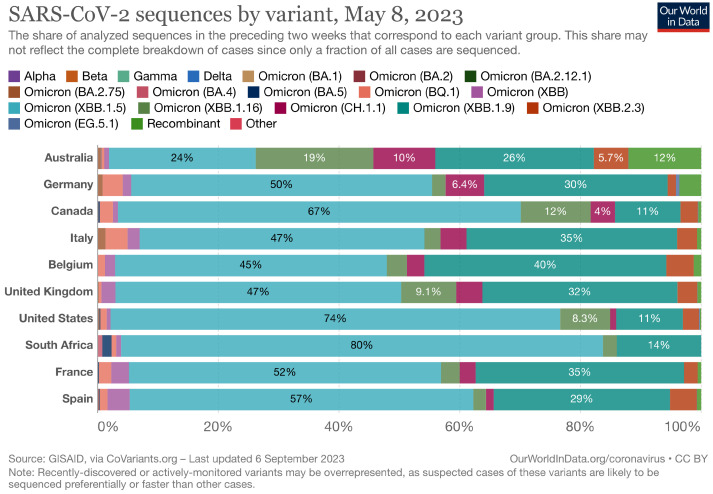
Relative frequency of Omicron variants in May. Image generated from https://ourworldindata.org/ (accessed on 29 January 2024) under open access under the Creative Commons BY license.

**Figure A4:**
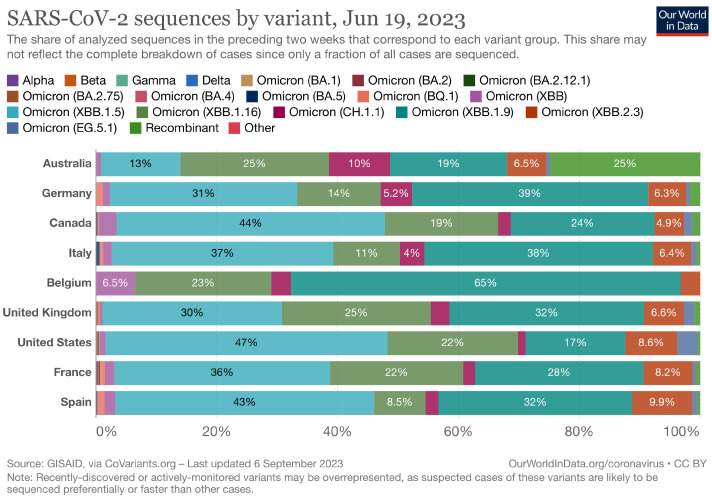
Relative frequency of Omicron variants in June. Image generated from https://ourworldindata.org/ (accessed on 29 January 2024) under open access under the Creative Commons BY license.

**Figure A5:**
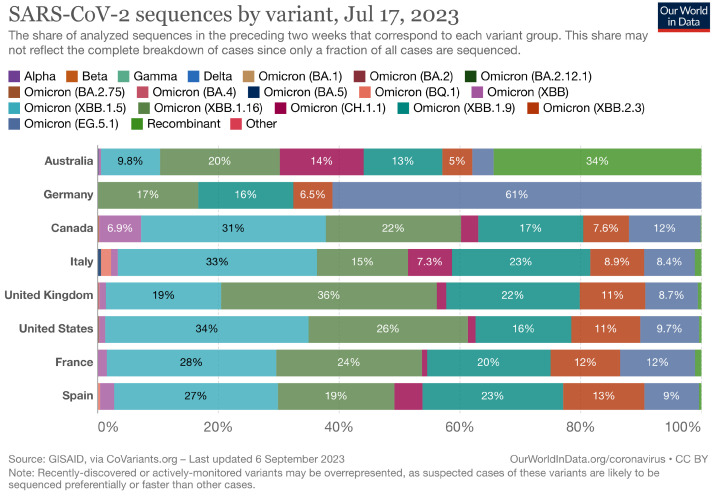
Relative frequency of Omicron variants in July. Image generated from https://ourworldindata.org/ (accessed on 29 January 2024) under open access under the Creative Commons BY license.

**Figure A6:**
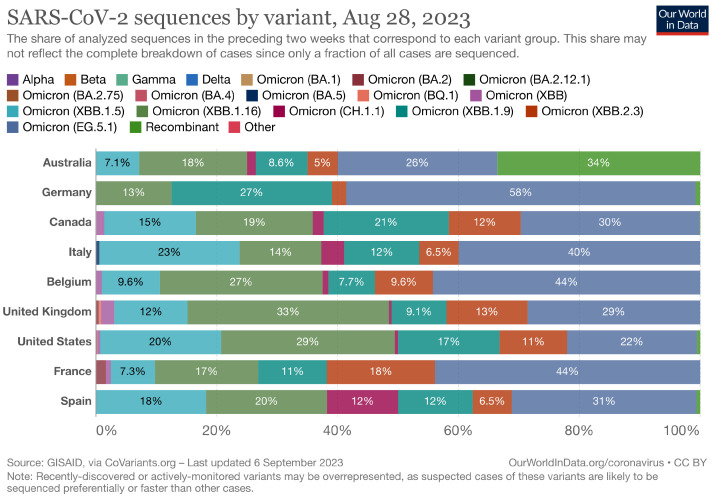
Relative frequency of Omicron variants in August 2023. Image generated from https://ourworldindata.org/ (accessed on 29 January 2024) under open access under the Creative Commons BY license.

## Data Availability

All the data and the code used for this paper are available at https://github.com/UgoLomoio/XBBSARSCoV2, (accessed on 29 January 2024). Third party software is available on their associated websites.

## Abbreviations

The following abbreviations are used in this manuscript:

ORF	Open reading frame
S	Spike
E	Envelope
M	Membrane
N	Nucleocapsid
WHO	World Health Organization
VBM	Variant Being Monitored
VOF	Variant of Concern
VOF	Variant of Concern
VOI	Variant of Interest
RBD	Receptor binding domain

## Appendix A

**Table A1:** This table shows which mutations are shared between variants Delta B1.167.2, Omicron BA.2, XBB.1.0, and EG5.1. Mutations “Q52H”, “F456L”, and “F486P” are present only in the mutation EG.5.1. Mutations “T376A”, “D405N”, and “R408S” are present in XBB, Omicron, and EG5.1 variants and are not present in the other branches. Even if there are other mutations in a similar condition, these ones have been indicated as relevant since they occur in the binding domain. Mutations such as “T478K” are present in XBB and EG.5.1 and in at least one of the other variants. Other mutations in similar condition as latter are “K417N”, “F456L”, “T478K”, “F486P”, and “H655Y”.

Mutation	Alpha B1.1.7	Gamma P.1	Beta B.1.351	Delta B.1.167.2	Omicron BA.2	XBB.1.0	EG.5.1
**L18F**		X	X				
**T19R**				X			
**T19I**					X	X	X
**T20N**		X					
**L24S**					X	X	X
**P26S**		X					
**DEL25/27**					X	X	X
**Q52H**							X
**DEL 69/70**	X						
**D80A**			X				
**V83A**						X	X
**D138Y**		X					
**G142D**				X		X	X
**DEL144/144**	X					X	X
**H146Q**						X	X
**E156G**				X			
**DEL157/158**				X			
**Q183E**						X	X
**R190S**		X					
**V213G**					X		
**V213E**						X	X
**D215G**			X				
**DEL 242/244**			X				
**R346I**			X				
**G252V**						X	X
**G339D**					X		
**G339H**						X	X
**R346T**						X	X
**L381I**						X	X
**S371F**					X	X	X
**S373P**					X	X	X
**S375F**					X	X	X
**T376A**					X	X	X
**D405N**					X	X	X
**R408S**					X	X	X
**K417N**			X		X	X	X
**N440K**					X	X	X
**V445P**						X	X
**G446S**						X	X
**L452R**				X			
**N460K**						X	X
**K417T**		X					
**S477N**					X	X	X
**T478K**				X	X	X	X
**F456L**							X
**E484A**					X	X	X
**E484K**		X	X				
**F486P**							X
**F490S**						X	X
**Q493R**					X		
**Q498R**					X	X	X
**N501Y**	X	X	X		X	X	X
**Y505H**					X	X	X
**A570D**	X						
**D614G**	X	X	X	X	X	X	X
**H655Y**		X			X	X	X
**N679K**					X	X	X
**P681H**	X				X	X	X
**P681R**				X			
**A701V**			X				
**T716I**	X						
**N764K**					X	X	X
**D796Y**					X	X	X
**D950N**				X			
**Q954H**					X	X	X
**N969K**					X	X	X
**S982A**	X						
**T1027Y**		X					
**V1176F**		X					
**D1118H**	X						

**Table A2:** Gibbs free energy associated with each single mutation. Changes in Gibbs free energy impact the binding affinity of the spike protein to the ACE2 receptor.

Mutation	Δ *G*	Variants
WT (PDB: 7DF4)	−13.6	
D405N	−13.9	Omicron (BA.2), XBB.1.0,
		EG.5.1
D614G	−13.6	Alpha (B1.1.7), Gamma (P.1), Beta (B.1.351), Delta (B.1.167.2), Omicron (BA.2), XBB.1.0,
		EG.5.1
F456L	−13.5	EG.5.1
F486P	−13.4	EG.5.1
H655Y	−13.6	Gamma (P.1), Omicron (BA.2), XBB.1.0
		EG.5.1
K417N	−13.7	Beta (B.1.351), Omicron (BA.2), XBB.1.0
		EG.5.1
N501Y	−13.3	Alpha (B1.1.7), Gamma (P.1), Beta (B.1.351), Omicron (BA.2), XBB.1.0,
		EG.5.1
P681H	−13.6	Alpha (B1.1.7), Omicron (BA.2), XBB.1.0
		EG.5.1
Q52H	−13.6	EG.5.1
R408S	−13.7	Omicron (BA.2), XBB.1.0,
		EG.5.1
T376A	−13.6	Omicron (BA.2), XBB.1.0,
		EG.5.1
T478K	−13.6	Delta (B.1.167.2), Omicron (BA.2), XBB.1.0,
		EG.5.1

## References

[1] Guzzi P.H., Mercatelli D., Ceraolo C., Giorgi F.M. (2020). Master regulator analysis of the SARS-CoV-2/human interactome. J. Clin. Med..

[2] Kim D., Lee J.Y., Yang J.S., Kim J.W., Kim V.N., Chang H. (2020). The architecture of SARS-CoV-2 transcriptome. Cell.

[3] Kumar Das J., Tradigo G., Veltri P., Guzzi P.H., Roy S. (2021). Data science in unveiling COVID-19 pathogenesis and diagnosis: Evolutionary origin to drug repurposing. Briefings Bioinform..

[4] Domingo E., Holland J. (1997). RNA virus mutations and fitness for survival. Annu. Rev. Microbiol..

[5] Madhi S.A., Kwatra G., Myers J.E., Jassat W., Dhar N., Mukendi C.K., Nana A.J., Blumberg L., Welch R., Ngorima-Mabhena N. (2022). Population immunity and COVID-19 severity with Omicron variant in South Africa. N. Engl. J. Med..

[6] Lauring A.S., Frydman J., Andino R. (2013). The role of mutational robustness in RNA virus evolution. Nat. Rev. Microbiol..

[7] Wu S., Tian C., Liu P., Guo D., Zheng W., Huang X., Zhang Y., Liu L. (2021). Effects of SARS-CoV-2 mutations on protein structures and intraviral protein–protein interactions. J. Med. Virol..

[8] Boni M.F., Lemey P., Jiang X., Lam T.T.Y., Perry B.W., Castoe T.A., Rambaut A., Robertson D.L. (2020). Evolutionary origins of the SARS-CoV-2 sarbecovirus lineage responsible for the COVID-19 pandemic. Nat. Microbiol..

[9] Tournier J.D., Mori S., Leemans A. (2011). Diffusion tensor imaging and beyond. Magn. Reson. Med..

[10] Oude Munnink B.B., Worp N., Nieuwenhuijse D.F., Sikkema R.S., Haagmans B., Fouchier R.A., Koopmans M. (2021). The next phase of SARS-CoV-2 surveillance: Real-time molecular epidemiology. Nat. Med..

[11] Hiram Guzzi P., Petrizzelli F., Mazza T. (2022). Disease spreading modeling and analysis: A survey. Briefings Bioinform..

[12] Hu B., Guo H., Zhou P., Shi Z.L. (2021). Characteristics of SARS-CoV-2 and COVID-19. Nat. Rev. Microbiol..

[13] Guzzi P.H., Di Paola L., Puccio B., Lomoio U., Giuliani A., Veltri P. (2023). Computational analysis of the sequence-structure relation in SARS-CoV-2 spike protein using protein contact networks. Sci. Rep..

[14] Burki T.K. (2022). Omicron variant and booster COVID-19 vaccines. Lancet Respir. Med..

[15] Du Z., Hong H., Wang S., Ma L., Liu C., Bai Y., Adam D.C., Tian L., Wang L., Lau E.H. (2022). Reproduction number of the omicron variant triples that of the delta variant. Viruses.

[16] Tamura T., Ito J., Uriu K., Zahradnik J., Kida I., Anraku Y., Nasser H., Shofa M., Oda Y., Lytras S. (2023). Virological characteristics of the SARS-CoV-2 XBB variant derived from recombination of two Omicron subvariants. Nat. Commun..

[17] Zhou Y., Wang F., Tang J., Nussinov R., Cheng F. (2020). Artificial intelligence in COVID-19 drug repurposing. Lancet Digit. Health.

[18] Yamasoba D., Uriu K., Plianchaisuk A., Kosugi Y., Pan L., Zahradnik J., Ito J., Sato K. (2023). Virological characteristics of the SARS-CoV-2 Omicron XBB. 1.16 variant. Lancet Infect. Dis..

[19] Scarpa F., Sanna D., Azzena I., Casu M., Cossu P., Fiori P.L., Benvenuto D., Imperia E., Giovanetti M., Ceccarelli G. (2023). Genome-based comparison between the recombinant SARS-CoV-2 XBB and its parental lineages. J. Med. Virol..

[20] Uriu K., Ito J., Zahradnik J., Fujita S., Kosugi Y., Schreiber G., Sato K. (2023). Enhanced transmissibility, infectivity, and immune resistance of the SARS-CoV-2 omicron XBB. 1.5 variant. Lancet Infect. Dis..

[21] Yuan M., Huang D., Lee C.C.D., Wu N.C., Jackson A.M., Zhu X., Liu H., Peng L., Van Gils M.J., Sanders R.W. (2021). Structural and functional ramifications of antigenic drift in recent SARS-CoV-2 variants. Science.

[22] Elbe S., Buckland-Merrett G. (2017). Data, disease and diplomacy: GISAID’s innovative contribution to global health. Glob. Chall..

[23] Hansen C.H., Friis N.U., Bager P., Stegger M., Fonager J., Fomsgaard A., Gram M.A., Christiansen L.E., Ethelberg S., Legarth R. (2023). Risk of reinfection, vaccine protection, and severity of infection with the BA. 5 omicron subvariant: A nation-wide population-based study in Denmark. Lancet Infect. Dis..

[24] Zhang Y., Skolnick J. (2004). Scoring function for automated assessment of protein structure template quality. Proteins Struct. Funct. Bioinform..

[25] Zhang C., Shine M., Pyle A.M., Zhang Y. (2022). US-align: Universal structure alignments of proteins, nucleic acids, and macromolecular complexes. Nat. Methods.

[26] Thompson J.D., Gibson T.J., Higgins D.G. (2003). Multiple sequence alignment using ClustalW and ClustalX. Curr. Protoc. Bioinform..

[27] Bittrich S., Rose Y., Segura J., Lowe R., Westbrook J.D., Duarte J.M., Burley S.K. (2022). RCSB Protein Data Bank: Improved annotation, search and visualization of membrane protein structures archived in the PDB. Bioinformatics.

[28] DeLano W.L. (2002). Pymol: An open-source molecular graphics tool. CCP4 Newsl. Protein Crystallogr..

[29] Xue L.C., Rodrigues J.P., Kastritis P.L., Bonvin A.M., Vangone A. (2016). PRODIGY: A web server for predicting the binding affinity of protein–protein complexes. Bioinformatics.

[30] Olsson M.H.M., Søndergaard C.R., Rostkowski M., Jensen J.H. (2011). PROPKA3: Consistent Treatment of Internal and Surface Residues in Empirical pKa Predictions. J. Chem. Theory Comput..

[31] Markosian C., Staquicini D.I., Dogra P., Dodero-Rojas E., Lubin J.H., Tang F.H., Smith T.L., Contessoto V.G., Libutti S.K., Wang Z. (2022). Genetic and Structural Analysis of SARS-CoV-2 Spike Protein for Universal Epitope Selection. Mol. Biol. Evol..

[32] Benjamini Y. (2010). Discovering the False Discovery Rate. J. R. Stat. Soc. Ser. Stat. Methodol..

[33] Wilcoxon F. (1998). Individual Comparisons by Ranking Methods. Breakthroughs in Statistics: Methodology and Distribution.

[34] Xia S., Wang L., Jiao F., Yu X., Xu W., Huang Z., Li X., Wang Q., Zhu Y., Man Q. (2023). SARS-CoV-2 Omicron subvariants exhibit distinct fusogenicity, but similar sensitivity, to pan-CoV fusion inhibitors. Emerg. Microbes Infect..

[35] Ciccozzi M., Pascarella S. (2023). Two sides of the same coin: The N-terminal and the receptor-binding domains of SARS-CoV-2 Spike. Future Virol..

[36] Jalali N., Brustad H.K., Frigessi A., MacDonald E.A., Meijerink H., Feruglio S.L., Nygård K.M., Rø G., Madslien E.H., De Blasio B.F. (2022). Increased household transmission and immune escape of the SARS-CoV-2 Omicron compared to Delta variants. Nat. Commun..

[37] Uraki R., Ito M., Kiso M., Yamayoshi S., Iwatsuki-Horimoto K., Furusawa Y., Sakai-Tagawa Y., Imai M., Koga M., Yamamoto S. (2023). Antiviral and bivalent vaccine efficacy against an omicron XBB. 1.5 isolate. Lancet Infect. Dis..

[38] Conforti C., Dianzani C., Agozzino M., Giuffrida R., Marangi G.F., di Meo N., Morariu S.H., Persichetti P., Segreto F., Zalaudek I. (2020). Cutaneous manifestations in confirmed COVID-19 patients: A systematic review. Biology.

[39] Fayad N., Abi Habib W., Kandeil A., El-Shesheny R., Kamel M.N., Mourad Y., Mokhbat J., Kayali G., Goldstein J., Abdallah J. (2021). SARS-CoV-2 variants in Lebanon: Evolution and current situation. Biology.

